# Whole blood storage time impairs clot strength with minimal change in functional fibrinogen concentration

**DOI:** 10.1111/trf.70150

**Published:** 2026-03-11

**Authors:** Elizabeth R. Maginot, Nicolle K. Barmettler, Flobater I. Gawargi, Collin M. White, Natasha Goodman, Timothy R. Billiar, Refael Munitz, Scott A. Koepsell, Shelly M. Williams, Aleh Bobr, Phillip C. Spinella, Susan M. Shea, Reynold Henry, Christopher D. Barrett

**Affiliations:** 1 Division of Acute Care Surgery, Department of Surgery, University of Nebraska Medical Center, Omaha, Nebraska, USA; 2 Department of Cellular and Integrative Physiology, University of Nebraska Medical Center, Omaha, Nebraska, USA; 3 Department of Surgery, University of Pittsburgh Medical Center, Pittsburgh, Pennsylvania, USA; 4 Trauma and Transfusion Medicine Research Center, Department of Surgery, University of Pittsburgh, Pennsylvania, USA; 5 Department of Pathology, Microbiology and Immunology, University of Nebraska Medical Center, Omaha, Nebraska, USA; 6 Department of Surgery, Division of Trauma, University of Pittsburgh Medical Center, Pittsburgh, Pennsylvania, USA

**Keywords:** fibrinogen stability, leukoreduction, ROTEM, whole blood storage

## Abstract

**Background:**

Whole blood (WB) is a preferred resuscitation strategy, yet how storage affects hemostasis remains unclear. Prior studies have shown decreased fibrinogen function during storage; however, these were limited by incomplete removal of platelet contributions. Additionally, leukoreduced (LR) versus non-leukoreduced (NLR) status in preserving fibrinogen remains unknown.

**Study Methods:**

WB units (*n* = 15, 7 NLR, 8 nLR) were evaluated at serial time points through day 35. ROTEM EXTEM was performed. Platelet-poor plasma (PPP) from WB at day 0, day 21, and day 35 underwent western blot for fibrinogen. Functional fibrinogen was measured using the Clauss assay after freeze–thaw cycles of WB PPP to minimize platelet contribution.

**Results:**

As WB aged, there was a progressive decrease in alpha angle and maximum clot firmness. Platelet count dropped sharply by day 7. Western blot demonstrated no evidence of fibrinogen degradation in either LR or NLR WB. Clauss fibrinogen concentrations were unchanged from day 0 to day 35, with a modest non-significant decline by day 35 driven primarily by NLR units.

**Discussion:**

Fibrinogen did not undergo detectable proteolysis during storage of WB and had preserved function through day 35 after freeze–thaw cycles to minimize platelet contributions. NLR appeared to impact fibrinogen function with medians below 200 for day 21 and 35 but was not statistically significant. This suggests that previously reported reductions in fibrinogen function may be based on leukoreduction status, but that early changes in fibrinolysis sensitivity are more likely due to residual platelet contributions to the assays rather than loss of fibrinogen function itself.

## 1 | INTRODUCTION

Whole blood for hemostatic resuscitation has re-emerged as a preferred strategy in both military and civilian trauma care.^1–4^ Compared to component therapy, whole blood offers logistic simplicity and delivers a hemostatic product that resembles native circulating blood and avoids the dilutional effects inherent to reconstituted products.^1,4^ Contemporary studies have associated whole blood transfusion with reduced mortality, improved hemostatic profiles, and decreased transfusion requirements in severely injured trauma patients.^1,2,4–6^ As its use expands, a more comprehensive understanding of how storage and processing influence the hemostatic functional performance of whole blood is increasingly important.^5,6^

Fibrinogen is central to clot formation and hemostasis, yet several storage-related changes raise concern about its functional stability within whole blood units.^7,8^ Recent studies have demonstrated that stored whole blood becomes progressively more sensitive to tissue plasminogen activator (tPA), and that alpha-angle and maximum amplitude/maximum clot firmness (MCF) decline over time on viscoelastic assays—patterns consistent with impaired platelet and fibrinogen function.^8,9^ Importantly, these storage-related changes occur independent of patient physiology and therefore reflect intrinsic alterations within the blood product itself.^9^ In contrast, during states of trauma and hemorrhagic shock, endogenous tPA is released in large quantities, driving pathologic fibrinolytic phenotypes including hyperfibrinolysis.^10–12^ The interaction between these two processes, storage related sensitization of whole blood to tPA and trauma-induced elevations in circulating tPA at the time of transfusion, may have important clinical implications necessitating further study. Specifically, transfusion of older whole blood units that are more vulnerable to tPA-mediated fibrinolysis into patients experiencing high endogenous tPA release could create a “double-hit” scenario, potentially limiting the ability of the transfused blood to restore effective hemostasis, although in vivo studies are needed to corroborate this hypothesis.^9^

With regards to fibrinogen function specifically, these studies and others have methodologically failed to adequately consider or address possible confounding contributions of platelets to how fibrinogen function is measured. Platelets play a central role in hemostasis and clot stability through multiple antifibrinolytic mechanisms. They provide the primary binding site for fibrinogen via integrin *α*_IIB_*β*_3_, providing fibrinogen-platelet bridging that promotes initial platelet aggregation and supports early fibrin network formation.^13^ In addition, platelets contain the majority of circulating plasminogen activator inhibitor-1 (PAI-1), which is released upon platelet activation in response to thrombin and is a major determinant of resistance to fibrinolysis.^14,15^ Platelets are also an abundant source of factor XIII-A, which is essential for fibrin stabilization through crosslinking *α*2-antiplasmin to fibrin, limiting plasmin-mediated clot degradation.^14,16^ These underlying mechanisms highlight the multiple platelet-fibrin effects that contribute to viscoelastic values. Specifically, *α*-angle is sensitive not only to fibrin polymerization but also to platelet-fibrin interactions and factor XIII mediated-cross linking.^17,18^ In the study by Chae et al., for example, FIBTEM was used, which does not fully eliminate platelet contributions to fibrinogen function measurements.^8^ Similarly, fibrinogen function assessed by the Clauss method with fresh platelet-poor plasma (PPP) may still retain residual platelet contributions in the absence of a secondary method to remove residual platelet function (e.g. a freeze–thaw cycle, rather than using freshly made PPP).^8,19,20^ Thus, whether the reported reduction in fibrinogen function during whole blood storage is real, or merely a reflection of loss of platelet function over time during storage in combination with inadequate methods to remove platelet contributions from fibrinogen function measurements remains a critical question.

In addition to incomplete understanding of fibrinogen function in stored whole blood, other factors also require further consideration, particularly the presence or absence of leukocytes. Leukoreduction of whole blood may attenuate exposure to proteases, oxidative stress and inflammatory mediators released by leukocytes during storage.^21–24^ For example, it is known that fibrinogen loses its “knob” via neutrophil elastase cleavage of A*α*1-21 that removes the thrombin cleavage site and renders it incapable of linear polymerization (clot formation).^21–24^ Whether these processes meaningfully impact fibrinogen structure or function during whole blood storage, however, is not well studied. The work by Sivertson et al. did attempt to evaluate the impact of leukoreduction on hemostatic properties, but did not address the potential confounding roles of platelets discussed above,^19^ and thus the influence of leukoreduction to any change in fibrinogen function over time during whole blood storage remains incompletely understood.

To help address these knowledge gaps and the limitations of the prior literature, we performed a study to evaluate fibrinogen function with greater specificity as well as proteolytic integrity in both leukoreduced (LR) and non-leukoreduced (NLR) whole blood units over their storage duration obtained from a large, urban US blood bank. We hypothesized that (1) fibrinogen may undergo proteolytic degradation and/or loss of function over time in NLR whole blood, and (2) that LR whole blood would retain fibrinogen chain lengths (i.e., not degrade) and would not lose fibrinogen function over time.

## 2 | METHODS

### 2.1 | Whole blood samples and storage

Whole blood units (*n* = 15 total, *n* = 7 NLR, *n* = 8 LR) were collected prospectively from healthy volunteer donors at the University of Pittsburgh (IRB#23090051). All units were initially collected in citrate–phosphate-dextrose-adenine (CPDA-1) anticoagulant and stored at 4°C, with LR units having the additional step of being run through a platelet sparing leukoreduction filter prior to storage. The whole blood units were collected between October 2023 and December 2024. Each unit was analyzed for fibrinogen function and proteolysis/degradation at three different predefined storage timepoints (early: day 0; mid: day 21; late: day 35), allowing for intraunit comparisons over time. No repeated donations from the same donor were included. PPP was prepared by centrifuging whole blood at 3000 × gravity for 15 min followed by collection of the supernatant plasma, and repeat centrifugation with the top fraction of this taken as PPP. Aliquots of each whole blood sample were serially centrifuged (as above) at each timepoint for PPP collection and stored frozen at −80°C until use in assays.

### 2.2 | Rotational thromboelastometry

Whole blood samples were analyzed using rotational thromboelastometry (ROTEM) at four storage timepoints: day 0, day 7, day 10, day 21, and day 35. Functional clot integrity and extrinsic tissue factor-mediated coagulation were evaluated using extrinsically activated test (EXTEM) values of clotting time (CT), clot formation time, maximum clot firmness (MCF), alpha angle (AA), and lysis index at 60 minutes (LI60).

### 2.3 | Western blot of whole blood plasma for fibrinogen and degradation fragments

Western blot analysis was performed on PPP from whole blood samples at the earliest (day 0), mid (day 21) and late (day 35) time points to evaluate for proteolytic degradation during storage according to the method of Towbin (Figure 3, Supplemental Figure S2).^25^ Plasma samples were diluted 1:100 and run under reducing conditions to ensure any proteolysis would not be masked by disulfide bonds, and probed with a DAKO polyclonal rabbit anti-fibrinogen antibody at 1:75,000. Additionally, for positive control experiments, purified human fibrinogen (1 mg/mL, ProLytix) was incubated with purified human neutrophil elastase (Bio-Rad) at a concentration of 0.5 μg/mL for 4 h at 37°C and sampled over time. This elastase concentration was selected empirically to generate reproducible, partial fibrinogen degradation with clearly detectable lower-molecular weight fragments, while preserving intact chains for comparison and is markedly lower than levels generated by leukocytes when maximally stimulated such as in severe trauma.^26^ (Supplemental Figure S1). LI-COR fluorescent (IRDye 800CW) anti-rabbit IgG secondary antibody was used at 1:10,000 dilution for detection on a LI-COR Odyssey F4 imaging system (LI-COR Inc., Lincoln, NE).

### 2.4 | Clauss assay for functional fibrinogen concentration

Functional fibrinogen levels of whole blood were measured using a Clauss-based quantitative fibrinogen assay (QFA) using PPP that was frozen at −80°C immediately after isolation from whole blood until time of assay. Samples collected at different storage time points were frozen to allow for batched testing and were subsequently thawed and analyzed together for days 0 and 21, while day 35 was done separately. Importantly, the freeze–thaw cycle required to batch the samples together served as a mechanism to ensure minimal platelet contributions were made to Clauss assay results for functional fibrinogen concentrations. Compared to platelets, fibrinogen is stable across freeze–thaw cycles, with prior studies demonstrating minimal impact on fibrinogen concentration or function across variants.^27^ Samples collected at early (day 0), mid (day 21) and late (day 35) time points were tested, which was performed by our institutional core clinical laboratory on the Werfen Automated Coagulation Laboratory (ACL TOP 750) platform using the manufacturer’s standardized QFA protocol.

For each sample, PPP was automatically diluted by the analyzer and mixed with an excess concentration of bovine thrombin reagent (HemosIL QFA Thrombin, PN 0020301700). Under these conditions, thrombin is not rate-limiting; therefore, the time to fibrin clot formation depends solely on the concentration of functional fibrinogen present. Clot formation was optically detected by the instrument and converted into fibrinogen concentration (mg/dL) using a log–log calibration curve generated from reference calibrator plasma. Normal and low fibrinogen controls were run according to laboratory quality procedures to verify assay performance.

### 2.5 | Statistical analysis

All statistical analyses were performed using GraphPad Prism version 10 (GraphPad Software, San Diego, CA). An a priori power analysis was conducted using a coefficient of variation of 10% for fibrinogen functional assays. To detect a 25% difference between groups with an α of 0.05 and 80% power, a minimum of three samples per group was required. Comparison of hematologic and ROTEM parameters across storage intervals was performed using two-way ANOVA, to account for some missing data at the day 35 sample timepoint (5/15 samples missing at day 35 timepoint). Clauss assays were compared via Wilcoxon matched-pairs signed rank tests for functional fibrinogen concentrations between day 0 and day 21, while Mann–Whitney U-test was used to compare day 0 and 35 as sample limitations led to some missing values in the day 35 samples (5/15 samples missing: *n* = 6 included in LR, *n* = 4 included in NLR). A *p*-value <.05 was considered statistically significant.

## 3 | RESULTS

### 3.1 | Sample characteristics and demographics

A total of 15 whole blood samples were assessed over storage time (*n* = 7 NLR, *n* = 8 LR). Donor demographics are shown in Table 1. The median age was 32 years [IQR 21.8–36.3], and 35.7% were male. The median BMI was 24 kg/m^2^ [IQR 20.4–30.6]. All major ABO blood types were represented, with the majority of donors being O-Positive (42.9%).

### 3.2 | Longer storage time leads to reduced clot strength

**FIGURE 1:**
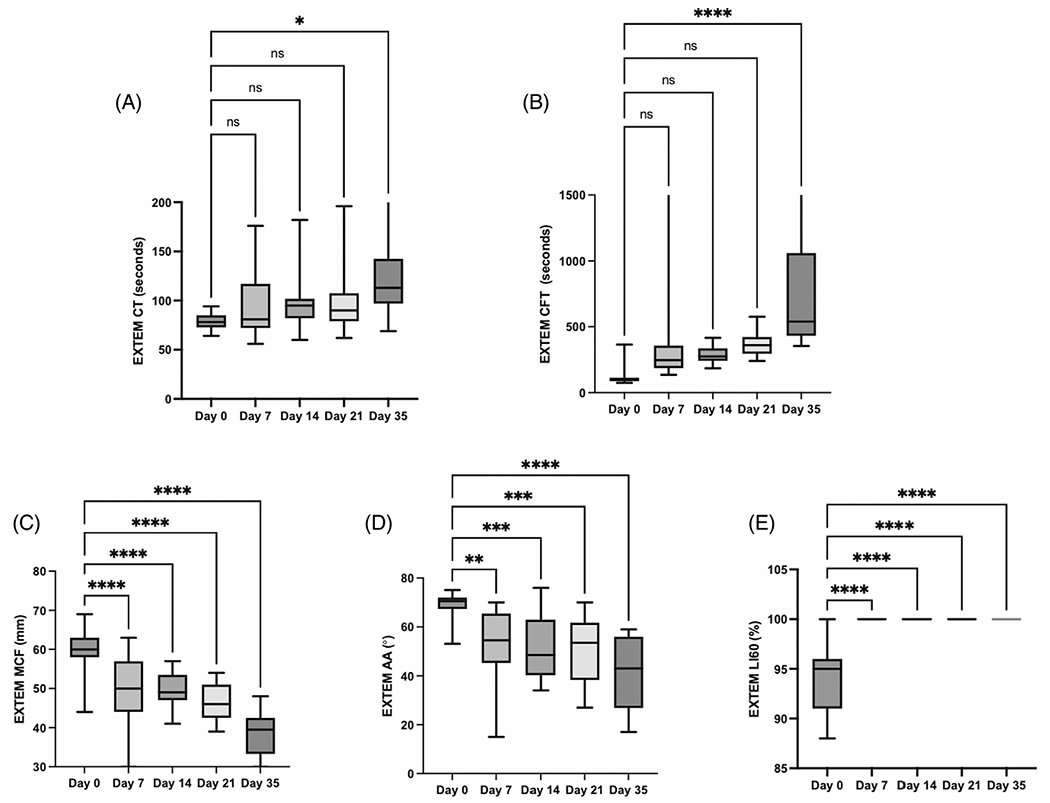
ROTEM EXTEM parameters decline with storage time. EXTEM clot parameters demonstrated progressive impairment in clot formation and strength over the storage period. (A) Clotting time (CT) increased by day 7. (B) Alpha angle (AA) and (C) maximum clot firmness (MCF) declined steadily from day 0 through 21. (D) Fibrinolysis decreased over time, with LI 60 reaching 100% by day 7. Data represent individual whole blood units with median values shown (*p < 0.05, **p < 0.01, ***p < 0.001, ****p < 0.0001).

**FIGURE 2:**
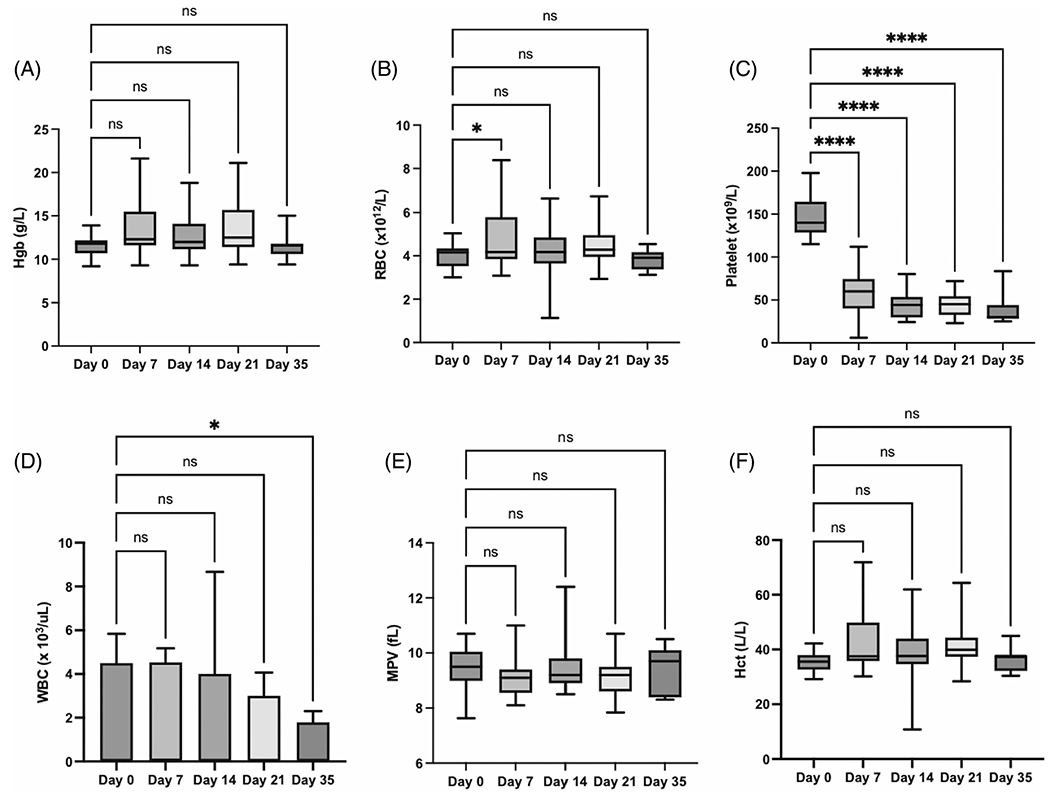
Routine hematologic parameters during cold storage. (C) Parallel laboratory values demonstrated stable (A) hemoglobin and (B) hematocrit over time, while there was a marked decline in platelet count beginning by day 7 (C). (D) White blood cell (WBC) count, (E) mean platelet volume, and (F) red cell indices were stable throughout storage. Points represent individual donor units with median values shown (*p < 0.05, **p < 0.01, ***p < 0.001, ****p < 0.0001).

With longer storage duration, we observed progressive impairment in clot formation and strength along with reduced endogenous (non-tPA challenged) fibrinolysis on ROTEM EXTEM assays (Figure 1). EXTEM AA and MCF both declined over time (all *p* <.01, Figure 1C,D). Similarly, clot strength was markedly reduced by these metrics, consistent with diminished fibrinogen and/or platelet function. Endogenous fibrinolytic activity was also absent by day 7 (Figure 1E). Overall, these findings suggest that the hemostatic potential of whole blood deteriorates with storage, likely due to loss of platelet function, reduced fibrin polymerization efficiency, or both. Parallel trends were also observed in routine hematologic laboratory parameters (Table 2, Figure 2). Platelet counts fell sharply by day 7 (*p* <.0001) and remained low thereafter, while hemoglobin, hematocrit, and red cell indices showed minimal change (Table 2, Figure 2). White blood cell count and mean platelet volume were also stable across time points. These data support that cellular integrity was largely preserved, except for platelets, which were lost early in storage and is consistent with the reduced EXTEM MCF observed at later storage time points (*p*<.0001).

### 3.3 | Fibrinogen does not undergo proteolysis in LR or NLR whole blood during storage

**FIGURE 3:**
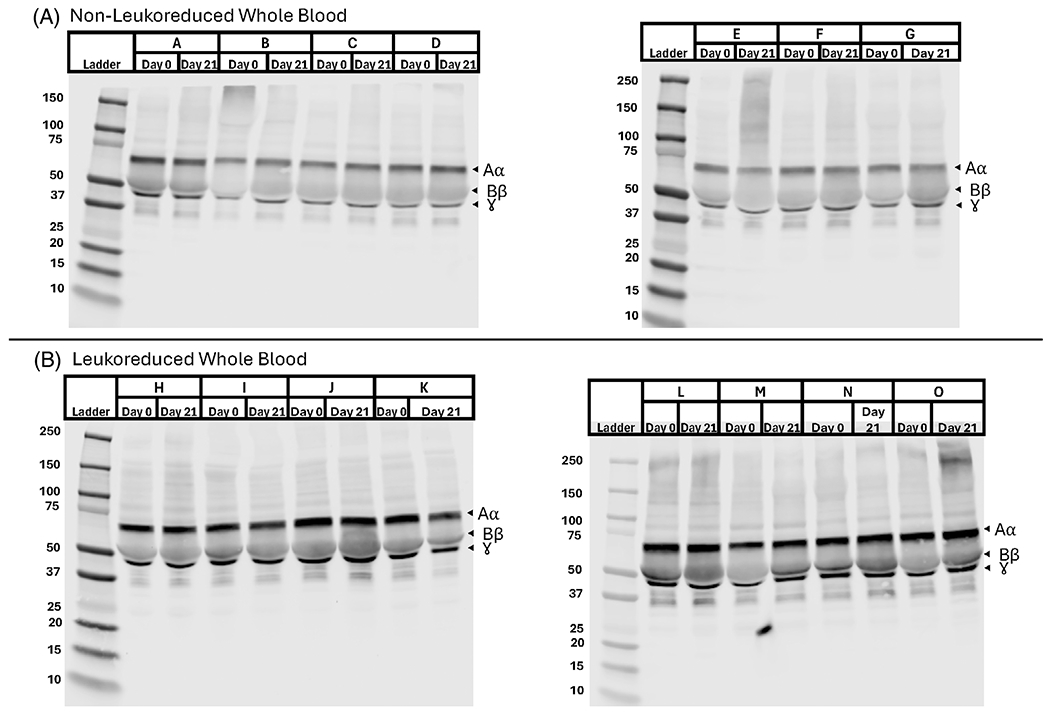
Fibrinogen does not undergo proteolytic degradation during storage. Western blot analysis of fibrinogen shows preserved molecular weights across storage time points of A*α*,B*β*, and *γ* fibrinogen chains for both (A) non-leukoreduced and (B) leukoreduced whole blood unit platelet-poor plasma.

Western blot analysis of both NLR and LR whole blood plasma showed no evidence of new or shifted band molecular weights, with preserved levels of full-length fibrinogen chains (A*α*, B*β*, and *γ* chains) that were similar in appearance across all whole blood units throughout the duration of storage (Figure 3, Supplemental Figure S2). In contrast, exposure of fibrinogen to leukocyte elastase led to marked shifts in molecular weights of the observed bands, likely representing smaller fibrinogen degradation products around 10–37 kDa (Supplemental Figure S1). This indicates that no detectable proteolysis of fibrinogen occurs during storage of whole blood and suggests that the decline in clot formation and strength observed on EXTEM over whole blood storage duration is not driven by proteolytic breakdown of fibrinogen in either LR or NLR whole blood.

### 3.4 | Functional fibrinogen concentration is mostly preserved during whole blood storage

**FIGURE 4:**
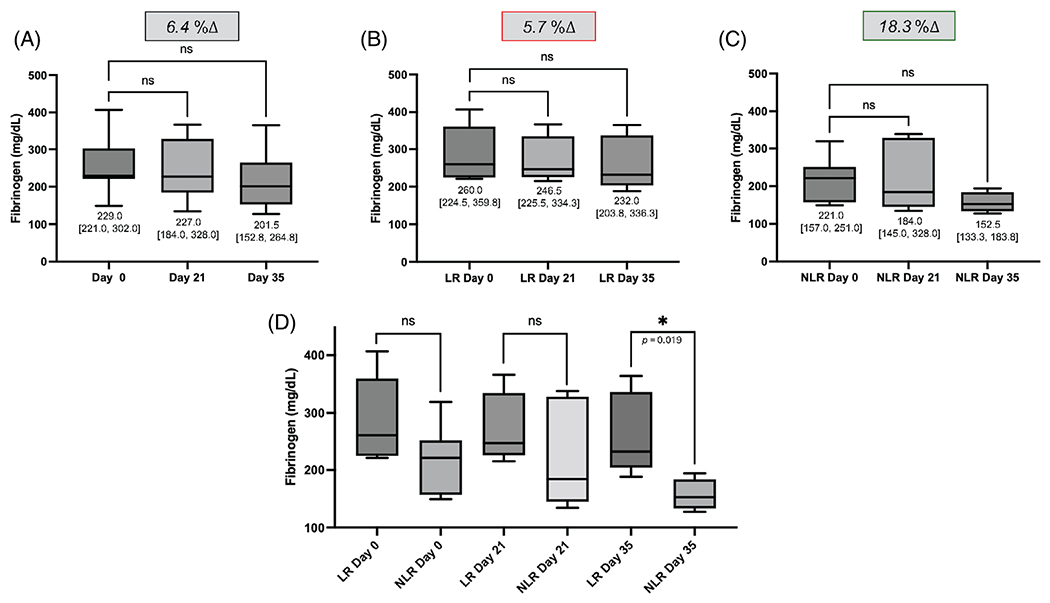
Functional fibrinogen concentration is preserved in leukoreduced units but appears to decline with time in non-leukoreduced units. (A) Clauss functional fibrinogen measurements demonstrated no significant change between day 0 and day 21 (*p* = .08) or day 0 and day 35 (*p* = .15), with an overall percentage change between day 0 and day 35 of 6.4%. (B) In leukoreduced (LR) units, functional fibrinogen levels showed a non-significant decline at days 21 (*p* = .06) and day 35 (*p* = .30), with an overall 5.7% reduction by day 35 and median values all remaining above the clinically relevant threshold of 200 mg/dL across all timepoints (day 0: 260 mg/dL; day 21: 246.5 mg/dL; day 35: 232.0 mg/dL). (C) In non-leukoreduced (NLR) units, fibrinogen levels showed a larger downward trend over time without reaching statistical significance (*p* = .71 from day 0 and day 21, *p* = .11 from day 0 to 35), but notably the median fibrinogen concentrations at day 21 and day 35 fell below 200 mg/dL (day 0: 221.0 mg/dL; day 21: 184.0 mg/dL; day 35: 152.5 mg/dL, respectively). (D) Direct comparison by leukoreduction status revealed a significant difference in functional fibrinogen concentration between LR and NLR units at day 35 (*p* = .019). (*p < 0.05, **p < 0.01, ***p < 0.001, ****p < 0.0001).

To further evaluate for any loss of fibrinogen function of LR and NLR whole blood during storage, we performed Clauss assays at day 0, day 21, and day 35 on PPP that all had been frozen at −80°C until time of assay, which served to remove platelet function from the residual platelets (Figure 4). In the overall cohort, functional fibrinogen concentration did not change significantly over time (Figure 4A), with a 6.4% overall (both LR and NLR combined) median percent change in functional fibrinogen concentration from day 0 to 35. When stratified by leukoreduction status, fibrinogen levels remained stable between day 0 to day 35 for LR samples (5.7% overall reduction by day 35), with all medians remaining above 200 mg/dL for all storage timepoints (Figure 4B). In the NLR samples, however, fibrinogen levels showed a downward trend of 18.3% by day 35, although this decrease was not statistically significant (*p* = .11) (Figure 2C). Importantly, in NLR the functional fibrinogen concentration was below 200 mg/dL at all times beyond day 0 (Figure 2C), and we did not observe a statistically significant difference in functional fibrinogen concentration between day 35 LR and day 35 NLR whole blood units (*p* = .019, Figure 4D). Taken together, these findings demonstrate no meaningful changes in fibrinogen function in LR whole blood out to day 35, while in NLR the finding is mixed with the appearance of a potentially meaningful decline by day 35 that did not reach statistical significance.

## 4 | DISCUSSION

This study expands upon our understanding of the hemostatic profile of whole blood, particularly as it relates to fibrinogen biology during storage. In a cohort of 15 whole blood donor units collected and processed through a major US blood bank, we observed a clear and progressive decline in clot formation and strength on ROTEM EXTEM assays. Both EXTEM AA and MCF decreased steadily across the storage period (Figure 1). While the literature has suggested a decline in fibrinogen function over time during whole blood storage, and these results were at least consistent with that possibility, it was just as likely that platelet death and/or platelet function loss was potentially responsible for both parameter changes. Therefore, to further investigate for any change in fibrinogen function, including differences between LR and NLR, we performed reducing western blots on plasma at day 0, day 21, and day 35 from both types of whole blood using a polyclonal anti-fibrinogen antibody to see if we could detect any proteolytic degradation (Figure 3, Supplemental Figures 1 and 2). As seen in Figure 3 and Supplemental Figure 2, however, we found preserved full-length A*α*, B*β*, and *γ* chains of fibrinogen throughout storage in both LR and NLR whole blood, with no evidence of lower molecular-weight fragments, indicating an absence of proteolytic fibrinogen breakdown in both LR and NLR whole blood during storage.

While proteolytic breakdown of fibrinogen was ruled out, that alone did not adjudicate a lack of fibrinogen function loss, as other post-translational modifications such as oxidative events, as well as defunctionalizing conformational changes/misfolding are also possible during storage. Therefore, we proceeded to evaluate functional fibrinogen concentrations over time utilizing the Clauss method, but importantly did so only after all whole blood derived PPP samples had been frozen at −80°C to minimize residual platelet contributions to the Clauss assay results while allowing temperature-stable fibrinogen to remain intact.^27^ This point is critical, as there are still small amounts of functional platelets present in fresh (unfrozen) PPP that can contribute, and other assays such as FIBTEM do not completely remove platelet contributions and thus cannot adequately inform loss of fibrinogen function during whole blood storage.^20^ As can be seen in Figure 4, no meaningful change in fibrinogen function was observed from day 0 to day 35 for the entire whole blood cohort together with a change of just 6.4% from day 0 to day 35 (Figure 4A).

When separating by leukoreduction status, in the LR samples fibrinogen levels gradually and non-significantly declined over time with just 5.7% loss by day 35, which was not statistically significant, and the median of all LR timepoints remained above 200 mg/dL, further suggesting this small change is unlikely to be clinically meaningful (Figure 4B). In contrast, the NLR samples’ functional fibrinogen levels showed a consistent downward trend (although not statistically significant in our cohort), and more importantly, the median functional fibrinogen levels at day 21 and day 35 fell below the clinically relevant threshold, measuring 184 mg/dL at day 21 and 152.5 mg/dL at day 35 (Figure 4C). When comparing LR to NLR units, the NLR units had a larger percent change from day 0 to day 35 compared to the LR units (18.3% and 5.7%, respectively) that was significantly different from each other on day 35 (Figure 4D). From a clinical perspective, these findings suggest that LR units would likely still support treatment of fibrinogen deficient states and retain the capacity to support fibrinolysis correction out to day 35 in CPDA-1, while in contrast older NLR units with fibrinogen levels well below 200 mg/dL may potentially contribute to impaired hemostasis.

These findings together are clinically important when considering the ideal expiration date of whole blood, especially based on leukoreduction status and the changing biology over storage time. Current evidence suggests that optimal hemostatic function is maintained through day 21, even though regulatory approval extends to 35 days in CPDA-1.^28,29^ At day 35, LR whole blood appeared similar to day 21, with no meaningful decline in fibrinogen function (Figure 4B). This stability likely also drove the overall combined data (LR + NLR) where no significant decline was observed (Figure 4A), which is highlighted by the intergroup day 35 comparisons (Figure 4D). In contrast, while not statistically significant on intragroup comparison, the NLR whole blood appeared to have a potentially clinically meaningful decline in fibrinogen function by days 21 and 35 (Figure 4C), with significantly lower fibrinogen function compared to LR at day 35 (Figure 4D), and proteolytic degradation by leukocyte proteases was largely ruled out as a cause by western blot analysis (Figures 3 and S2). Interpreting these findings in a clinically meaningful way further requires placing them in an in vivo context, where fibrinogen levels below 150–200 mg/dL are associated with an increased risk of bleeding in trauma patients and represent commonly used transfusion thresholds.^30,31^ This suggests that storage-related fibrinogen depletion in resuscitation products is variable based on the shelf-age and leukoreduction status of the product. This may also explain some of the variability observed across clinical trials of empiric fibrinogen supplementation in hemorrhaging trauma patients, although further study is needed to define these differences.^30,32^

While our data do not directly quantify residual platelet effects across assay preparations, they support the interpretation that previously reported reductions of fibrinogen function may, at least in part, reflect an artifact due to residual platelet contributions in the methodologies used. Notably, despite preservation of fibrinogen structure and function, we observed a marked decline in platelet count as early as day 7, with persistently reduced levels thereafter (Figure 2C). This is consistent with the well-described platelet storage lesion in whole blood and provides a plausible explanation for the progressive impairment in clot formation that we observed on viscoelastic testing, both in our study and prior reports (Figure 1).^9,17,19,33^ Platelets play a central role in hemostasis.^13,33^ They influence thrombin generation, fibrin polymerization kinetics, factor XIII-mediated cross-linking and resistance to fibrinolysis through platelet-fibrin interactions.^13,34,35^ As such, platelet loss and dysfunction during storage may exert a disproportionately large impact on overall hemostatic integrity, especially in the setting of preserved fibrinogen function through day 35 of stored whole blood. This study lays a foundation that storage-related declines in clot strength are more likely driven by platelet function rather than intrinsic fibrinogen function loss. Accordingly, future studies incorporating platelet function assays, factor XIII activity measurements, or platelet-fibrin interaction analyses are critical to further define the underlying biology of our observations and inform strategies aimed to optimize whole blood transfusion efficacy in trauma and hemorrhagic shock.

This study has several limitations. First, the donors and study represent a single-center, ex vivo analysis, so needs to be validated in an external population. Second, our study was a relatively small number of units (*n* = 15 total, *n* = 7 NLR, *n* = 8 LR), with fewer comparisons at day 35 due to sample exhaustion (*n* = 4 NLR, *n* = 6 LR), although our power analysis demonstrated that only 3 units per group were needed to detect a 25% drop in functional concentration (corresponding to a drop of ~100 g/dL fibrinogen concentration from baseline of otherwise healthy subjects^36^). Additionally, our study was performed ex vivo, so our observations may not fully reflect in vivo behavior following transfusion. Finally, our study only evaluated whole blood units collected and stored in CPDA-1, so our findings may not reflect what happens in other whole blood storage solutions.

In summary, whole blood stored in CPDA-1 under standard conditions does not appear to lose fibrinogen function out to day 35 in LR whole blood, while in NLR whole blood units stored out to 35 days there may be a decline in fibrinogen function with values routinely below the clinical threshold of 200 mg/dL. We did not find any evidence of proteolytic degradation of fibrinogen during storage, either in LR or NLR whole blood units, so any true loss of fibrinogen function in the NLR group likely reflects other defunctionalizing processes. Our results instead suggest that loss of alpha-angle on viscoelastic studies, as early as day 7, may be due to residual platelet contributions at early time points that were lost at later time points due to platelet death and/or loss of function, as these prior studies used freshly prepared PPP from whole blood. Overall, our findings emphasize the central role of platelet-fibrin interactions in maintaining whole-blood hemostatic function and suggest that storage-related platelet loss may underlie declining clot performance over time. These observations support further investigation into the use of whole blood earlier in its storage life or targeted adjuncts, such as platelet transfusion or antifibrinolytic therapy, particularly in settings where only older units are available, though hypothetical improved hemostasis due to transfusion of whole blood units with shorter storage duration requires further clinical investigation.

## Supplementary Material

FIG S1

FIG S2

Additional supporting information can be found online in the Supporting Information section at the end of this article.

## Figures and Tables

**TABLE 1:** Whole blood donor demographics (*n* = 15).

Variable	All patients *N* = 15 (%) or median [IQR]
Age (years)	32 [22, 34]

Male sex (%)	5 (35.7%)

BMI (kg/m^2^)	25.6 [20.4, 30.1]

Leukoreduced (%)	8 (53.3%)

Blood type	
A-Negative	3 (20.0%)
A-Positive	1 (6.7%)
B-Negative	1 (6.7%)
B-Positive	2 (13.3%)
O-Negative	1 (6.7%)
O-Positive	7 (46.7%)

**TABLE 2:** Routine laboratory values of non-leukoreduced measured in non-leukoreduced and leukoreduced whole blood units across storage timepoints.

	Non-leukoreduced (median [IQR]					Leukoreduced (median [IQR])					p-value
	Day 0	Day 7	Day 14	Day 21	Day 35	Day 0	Day7	Day 14	Day 21	Day 35	
Hemoglobin (g/L)	11 [10.3, 12.1]	13.2 [11.8, 16.7]	11.7 [10.9, 13.3]	13.25 [10.7, 15,3]	11.3 [10.7, 11.8]	11.9 [11.7, 12.1]	12.2 [11.7, 12.7]	12.2 [11.8, 14.2]	12.5 [12.1, 13.6]	11.7 [11.3, 11.8]	.67
RBCs (× 10^3^/μL)	3.7 [3.1, 4.2]	4.6 [4.0, 6.5]	3.8 [3.4, 4.8]	4.6 [3.3, 5.0]	3.4 [3.2, 4.2]	4.2 [3.9, 4.4]	4.2 [3.9, 4.6]	4.3 [4.0, 4.9]	4.2 [4.0, 4.8]	3.9 [3.8, 4.0]	.49
Platelets (× 10^9^/μL)	152 [129, 177]	60 [44, 77.3]	39 [30, 53.8]	41 [34, 50.3]	44.2 [44.1, 65]	140 [130.5, 152]	60 [44.5, 73]	48 [34.5, 53]	45 [35.3, 54]	28.5 [27.5, 29.8]	.61
WBCs (× 10^3^/μL)	4.5 [3.9, 4.9]	4.5 [4.0, 4.8]	4.0 [3.6, 4.6]	3.0 [1.5, 3.3]	1.91 [1.79, 2.15]	0 [0, 0]	0 [0, 0]	0 [0, 0]	0 [0, 0]	0 [0, 0]	<.001
MPV (fL)	9.8 [9.7, 10.4]	9.3 [9.1, 9.5]	9.5 [9.2, 9.9]	9.6 [9.2, 9.9]	8.4 [8.4, 9.7]	9 [8.9, 9.2]	8.6 [8.4, 9.1]	8.9 [8.8, 9.5]	9.1 [8.6, 9.2]	9.9 [9.7, 10.1]	.14
Hematocrit (L/L)	33.5 [30.4, 37.2]	39.7 [37.5, 55.4]	35.3 [29.6, 43.6]	42.4 [33.6, 43.9]	32.2 [31.1, 37.8]	35.7 [35.1, 37.5]	36.9 [36.0, 37.7]	38.4 [37.5, 43.1]	38.8 [38.7, 39.9]	37.7 [37.6, 37.8]	.57

*Note*: Data are presented as median with interquartile range. *p*-values reflect comparison between mean non-leukoreduced and mean leukoreduced groups across all time points.

## Data Availability

The data that support the findings of this study are available from the corresponding author upon reasonable request.

## Abbreviations:

AA: alpha angle
ACL TOP 750: Werfen Automated Coagulation Laboratory Platform
BMI: body mass index
CFT: clot formation time
CPDA-1: citrate-phosphate-dextrose-adenine-1
CT: clotting time
EXTEM: extrinsically activated test
FIBTEM: fibrin-based thromboestogram
Hct: hematocrit
Hgb: hemoglobin
IQR: interquartile range
LI60: lysis index at 60 minutes
LR: leukoreduced
MCF: maximum clot firmness
MPV: mean platelet volume
NLR: nonleukoreduced
PPP: platelet-poor plasma
RBC: red blood cell
ROTEM: rotational thromboelastometry
tPA: tissue plasminogen activator
WB: whole blood
WBC: white blood cell
