# Supplementary figures and images for: Whole blood storage time impairs clot strength with minimal change in functional fibrinogen concentration
Source: Transfusion. Author manuscript; Available in PMC 2026 Jul 14. (PMC13367526; doi:10.1111/trf.70150)

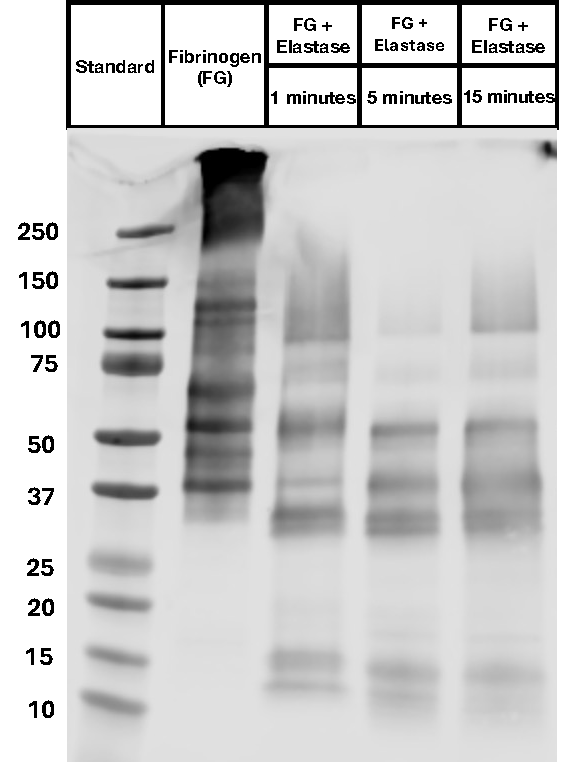

Supplement: FIG S1 [file NIHMS2188563-supplement-FIG_S1.tiff]

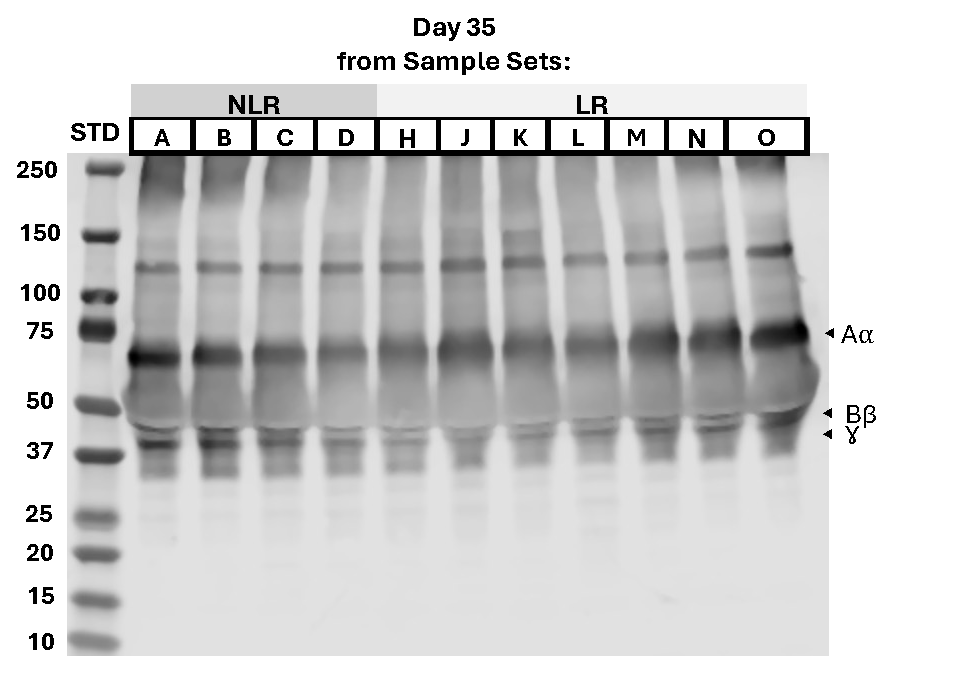

Supplement: FIG S2 [file NIHMS2188563-supplement-FIG_S2.tiff]
